# Can Orthopantomography be used as a tool for screening of carotid atheromatous pathology and thus be used to help reduce the prevalence of ischemic stroke within the population?

**DOI:** 10.4317/jced.50643

**Published:** 2012-02-01

**Authors:** Pedro Veiga Abecasis, Eduardo Chimenos-Küstner

**Affiliations:** 1 Assistente auxiliar, Instituto Superior de Ciências da Saúde-Egas Moniz- Departamento de Imagiologia; 2 Profesor titular de Medicina Bucal, Facultad de Odontología, Universidad de Barcelona

## Abstract

Objective: To assess the possibility of Dentists being able to screen patients with higher risk of vascular diseases. Material: Kodak 8000C Orthopantomographer, eco-Doppler Logiq-500 General Electric at the Lisbon Hospital Particular. Methods: Assessment of orthopantomographies made to 142 patients aged 50 or more, as well as the existing risk factors. Conduction of carotid eco-Doppler to patients who appear to have calcified plaques of the atheroma. Results: Strong dependence between dichotomised age and having the pathology (p = 0.02).Smokers are twice more likely to present plaques (OR= 2). Being hypertensive increases in about 1.4 the likelihood of having a stroke (OR= 1.4). Of the 27 individuals who presented calcifications in the Orthopantomography, they were all submitted to an eco-Doppler and 21 had the pathology confirmed. 27 individuals, who did not show any plaques in the Orthopantomography, were randomly selected to be the control group. They were submitted to an eco-Doppler. And 23 confirmed the non-existence of plaques. Conclusions: Orthopantomography used for assessing the oral cavity reveals more information which should be the object of the Dentist’s attention.

** Key words:**Orthopantomography, atheroma, stroke.

## Introduction

About 30% of stroke cases may be attributed to carotid bifurcation artherosclerotic lesions. About 75% of all strokes have an ischemic nature. (1)

Atherosclerosis of the carotid represent about 10 to 20% of the cases of stroke, which represent the third cause of death in the United States, with an incidence of 700,000 cases every year(2,3). Perhaps the most important aspect for the diagnosis of extracranial carotid stenosis is the suspicion of such condition through the identification of risk factors(2). Su and colleagues have studied the relation between systemic arterial hypertension and extracranial carotid stenosis in 263 hypertensive patients, comparing them to 270 normotensive patients. One may observe a 5 times increase in the occurrence of extracranial carotid stenosis in hypertensive patients when compared to normotensive patients (OR: 5, IC 95%)(4).

Smoking is an independent risk factor for strokes (it increases such risk in about 6 times). People who quit smoking reduce the risk of stroke in about 50% (5,6).

The carotid Doppler is a sensitive and specific method for diagnosing carotid stenosis, when compared to angiography, reaching sensitivity levels between 86% and 100%, and specificities between 82% and 94% (1).

 The Rotterdam Study showed that the population with calcified atheroma plaques had increased probabilities of suffering from myocardial infarction and stroke (7).

The FDA and the ADA recommend an Orthopantomography to every new edentulous patients due to the high prevalence (33%-41%) of discoveries such as root fragments and radio-transparent lesions (8). Dentists who use panoramic X-rays in their clinical practice as recommended by the ADA and the FDA should be able to observe all alterations and information with no dental origin or relation and thus ensure that patients are referred to the appropriate professionals (9).

In 1981, Friedlander and Lande (10) were the first to describe the panoramic X-Ray as a supplementary means for identifying patients at risk of suffering from strokes. By assessing a thousand panoramic X-Rays of male individuals aged between 50 and 75, they identified calcifications in the region of the bifurcation of the carotid artery in 2% of cases. Those images showed that 88% were calcifications of the carotid artery and the remaining 12% were calcified lymph nodes or salivary calculi (10).

In 2007 Friedlander (11) concluded that the most recent studies prove that patients with calcified atheromas in the carotid artery have an increased probability of suffering a nonfatal stroke. Thus, the accidental observation of calcifications in the carotid through an Orthopantomography becomes a powerful tool to screen patients with greater probabilities of suffering a myocardial infarction and other brain and cardiovascular pathologies (11).

## Material and Methods

Study design: Observational, analytical and transversal.

Population and dimension: 142 patients of the dental medicine service of the Lisbon Hospital Particular (HPL) who may have had an Orthopantomography in their first appointment.

Inclusion criteria: Patients from the Dental Medicine clinic over 50 years of age from both genders.

Exclusion criteria: Patients who have already been submitted to head or neck surgery; Patients without an Orthopantomography; Patients under 50 years of age.

Ethical evaluation: Study approved by the Ethics Committee of the Particular Hospital and all patients signed an informed permission.

Methodology

After planning every detail of the study, it starts with the analisys of the orthopantomographies to evaluate their quality and reject the incorrect ones where the spine does not figure. Then do the questionnaire with the patient. All 142 individuals in our sample had an Orthopantomography performed to them (regardless of already having had one made before) and for those who revealed carotid atheroma plaques between the C3-C4 vertebrae, an eco-Doppler was performed in order to confirm the pathology that had been detected.

27 individuals, who did not reveal plaques in the Orthopantomography, were then randomly selected to work as a control group. They were submitted to an eco-Doppler.

To assess the patients’ orthopantomographies made in the dental clinic of the Particular Hospital; to assess for each patient the presence of arterial hypertension diagnosed by the cardiologist, smoking habits and whether patients practice physical exercise with a complete questionnaire; Statistical assessment of results by using a significance level below 5% (p≤ 5%).

The tests used in this study was non-parametric tests; Mann Whitney; Test for quantitative variables, and Chi-square test for qualitative variables, plus the Odds Ratio.

Equipment used 

The Eco-Doppler of the HPL, model Logiq-500 General Electric; Exam conducted by the vascular surgeon Dr. Joaquim Barbosa.

The Orthopantomographer of the HPL, Kodak 8000C Digital Panoramic – Exam conducted by the researcher.

## Results

There is a strong dependence between dichotomising age and having the pathology (p= 0.02). We may add, by using the Odds Ratio, that being over 60 increases in about 6 times the likelihood of having carotid plaques ( Table 1).

**Table 1:**
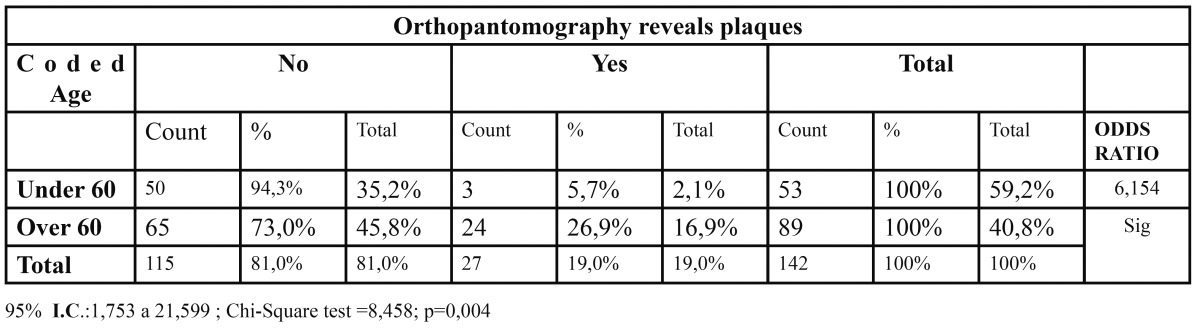
Relation between Coded Age and Orthopantomography revels plaques.

Smoking is a moderate risk factor, for smokers have twice the probability of having plaques OR=2 ( Table 2).

**Table 2:**
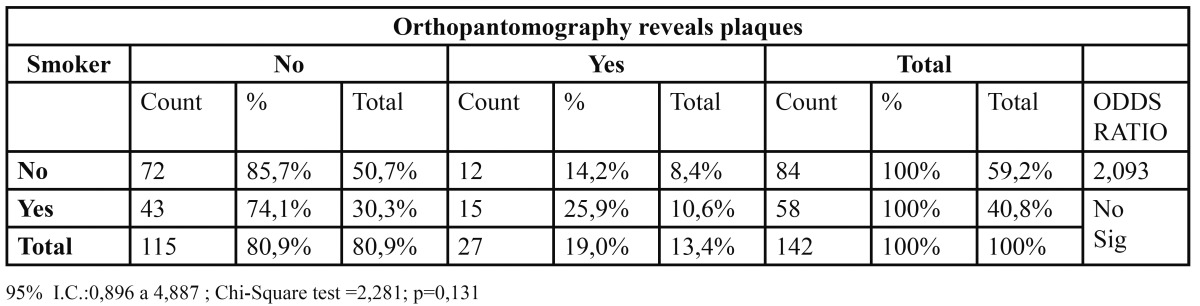
Relation between Smoker and Orthopantomography revels plaques.

Hypertension has little influence over the pathology under study. We may state that being hypertensive increases in about 1.4 times the likelihood of suffering a stroke (interpretation of the Odds Ratio) ( Table 3).

**Table 3:**
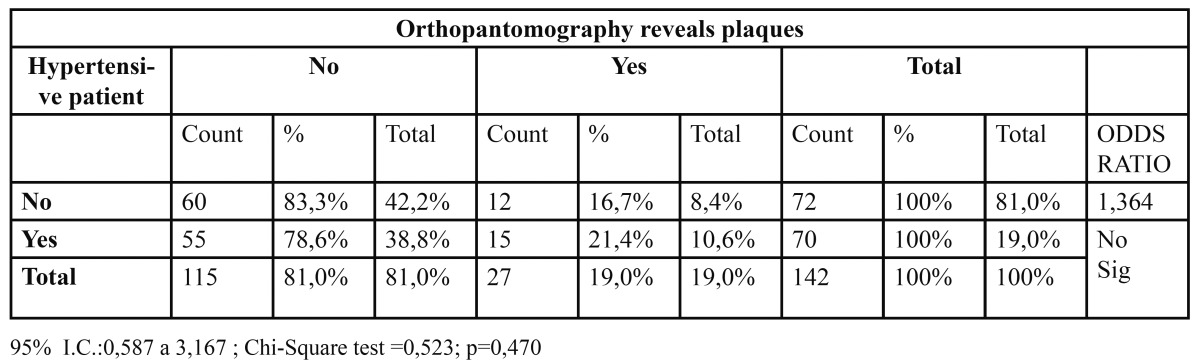
Relation between Hypertensive patient and Orthopantomography revels plaques.

Physical exercise represents a protective factor. In other words, those who do not exercise have 1.8 more probability of having carotid plaques. OR=0.57 ( Table 4).

**Table 4:**
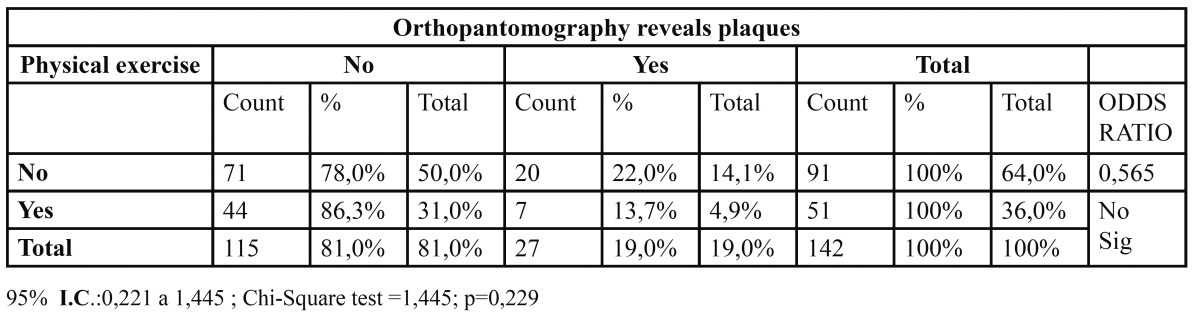
Relation between Practices Physical exercise and Orthopantomography revels plaques.

We used the Chi-square test as an independence test at all times (refer to the annex). However, only for matters related to age coded into two categories (<60 years and >60 years) did it reveal itself significant.

Out of the 27 individuals who presented calcifications in the carotid artery, the entire group was submitted to an eco-Doppler. 21 had their pathology confirmed. In other words, only 6 cases, 3 men and 3 women, did not reveal the pathology detected through the Orthopantomography.

27 individuals, who did not reveal plaques in the Orthopantomography, were then randomly selected to work as a control group. They were submitted to an eco-Doppler. And 23 confirmed the non-existence of plaques. In other words, only 4, all males, did not confirm the diagnosis based on the Orthopantomography.

By performing a Qui-Square test, using 27*2 =54 data to verify whether there is or not any concordance among the results, we can see that the “yes” and “no” percentages are very similar. In other words, there is no significant difference between the two groups in what the confirmation of the diagnosis done with the eco-Doppler is concerned. We may also state that the percentage of confirmed results is fairly high, that is to say, 78%.

## Discussion

Calcified atheroma plaques may be detected with panoramic X-rays as one or more adjacent nodular radio-opaque non-continuous images at the level of the C3 and C4 inter-vertebral joint (2,3,7,8,10).

As mentioned in the literature (8) sometimes the appearance of opaque images in the Orthopantomography may also reveal salivary calculi or calcified lymph nodes.

With this study we were able to confirm that age is a risk factor, as being over 60 increases in about 6 times the incidence of this pathology in and of itself.

Smoking, as mentioned in several articles, is also a risk factor which increases the incidence of cardiovascular disease (2,6,11).

In this study, and unlike others, hypertension, though associated to an increase of the carotid pathology, did not reveal itself as so significant, contributing to a 1.4 increased risk of occurrence of calcified plaques (2,4,11).

Physical exercise is a protective factor as for those who exercise it reduces the likelihood of developing atheromatous pathology (2,11).

When the eco-Doppler was randomly conducted within the control group, some patients were detected with arterial occlusion, thus showing that sometimes the atheroma plaques are not calcified and thus go unnoticed in the Orthopantomography (1,5,10,11).

One may thus confirm that it is actually possible to visualise, through a thorough analysis of Orthopantomographies properly taken, the presence of calcified atheroma plaques in the carotid artery (2,7,9,10,11).

So, it is an imperative to agree with the ADA’s scientific board which recommends that Dentists analyse and review the Orthopantomography seeking for evidence of atheromas. This attitude may have the potential to save lives. It does not however replace the eco-Doppler exam, but it increases the probabilities for screening and referral to a Neurologist (3).

We may conclude that the use of Orthopantomography brings several benefits to the planning and the general view of the oral cavity (2,7,8). And since we are having the exam for such purposes, why not make the most of all the information it has to offer us? As ischemic stroke is the cause of so many deaths, it may be concluded that, without any pretension to diagnose or treat this pathology, Dentists may at least be sensitive to these issues in their clinical practice and thus contribute towards the screening of arterial occlusion (2,3,7).

The purpose is not that Dentists start to diagnose or treat such pathology, but that they may be able to identify the risk and refer their patients to a specialist. Thus, several asymptomatic patients who do not consult a neurologist on their own initiative, may see their risk of developing severe and disabling thromboembolic scenarios significantly reduced with a simple eco-Doppler exam.
